# HIV-1 drug resistance testing is essential for heavily-treated patients switching from first- to second-line regimens in resource-limited settings: evidence from routine clinical practice in Cameroon

**DOI:** 10.1186/s12879-019-3871-0

**Published:** 2019-03-12

**Authors:** Desire Takou, Joseph Fokam, Georges Teto, Maria-Mercedes Santoro, Francesca Ceccherini-Silberstein, Aubin Joseph Nanfack, Samuel Martin Sosso, Béatrice Dambaya, Romina Salpini, Serge Clotaire Billong, Caterina Gori, Charles Ntungwen Fokunang, Giulia Cappelli, Vittorio Colizzi, Carlo-Federico Perno, Alexis Ndjolo

**Affiliations:** 1 Chantal BIYA International Reference Centre for research on HIV/AIDS prevention and management, Yaoundé, Cameroon; 2 Faculty of Medicine and Biomedical Sciences, University of Yaoundé I, Yaoundé, Cameroon; 3 National HIV Drug Resistance prevention and surveillance Working Group, Ministry of Public Health, Yaoundé, Cameroon; 4 University of Rome Tor Vergata, Rome, Italy; 5 Surveillance, Research, Planning, Monitoring and Evaluation service, Central Technical Group, National AIDS Control Committee, Yaounde, Cameroon; 6 National Institute of Infectious Diseases Lazzaro Spallanzani, Rome, Italy; 7 Institute of Cellular Biology and Neurobiology (IBCN), Consiglio Nazionale delle Ricerche (CNR), Rome, Italy; 8 University of Milan, Milan, Italy

**Keywords:** HIV drug resistance, Antiretroviral therapy, First-line, Protease, Reverse transcriptase, Cameroon

## Abstract

**Background:**

With the phase-out of stavudine (d4T), change to first-line regimens with zidovudine (AZT) or tenofovir (TDF) in resource-limited settings (RLS) might increase risks of cross-resistance to nucleos(t) ide reverse transcriptase inhibitors (NRTI). This would restrict the scope of switching to the World Health Organisation (WHO)-recommended standard second-line combinations (SLC) without HIV drug resistance (HIVDR)-testing in routine clinical practice.

**Methods:**

An observational study was conducted among 101 Cameroonian patients (55.4% male, median [IQR] age 34 [10–41] years) failing first-line antiretroviral therapy (ART) in 2016, and stratified into three groups according to NRTIs exposure: exposure to both thymidine analogues AZT “and” D4T (group-A, *n* = 55); exposure to both TDF and AZT “or” D4T (group-B, *n* = 22); exposure solely to D4T (group-C, *n* = 24). Protease-reverse transcriptase HIVDR was interpreted using the *HIVdb* penalty scores (≥60: high-resistance; 20–59: intermediate-resistance; < 20: susceptible). The acceptable threshold for potential-efficacy was set at 80%.

**Results:**

The median [IQR] CD4, viral RNA, and time on ART, were respectively 129 [29–466] cells/μl, 71,630 [19,041-368,000] copies/ml, and 4 [2–5] years. Overall HIVDR-level was 89.11% (90/101), with 83.2% harbouring M184 V (high-level 3TC/FTC-resistance) and only 1.98% (2/101) major HIVDR-mutations to ritonavir-boosted protease-inhibitors (PI/r). Thymidine-analogue mutations (TAMs)-1 [T215FY (46.53%), M41 L (22.77%), L210 W (8.91%)], with cross-resistance to AZT and TDF, were higher compared to TAMs-2 [D67N (21.78%), K70R (19.80%), K219QE (18.81%)]. As expected, K65R was related with TDF-exposure: 0% (0/55) in group-A, 22.72% (5/22) group-B, 4.17% (1/24) group-C (*p* = 0.0013). The potential-efficacy of AZT vs. TDF was respectively 43.64% (24/55) vs. 70.91% (39/55) in group-A (*p* = 0.0038); 63.64% (14/22) vs. 68.28% (15/22) in group-B (*p* = 1.0000); and 37.50% (9/24) vs. 83.33% (20/24) in group-C (*p* = 0.0032). CRF02_AG was the prevailing subtype (63.40%), followed by CRF11.cpx (8.91%), A_1_ (7.92%), G (5.94%); without any significant effect of the subtype-distribution on HIVDR (92.2% in CRF02_AG vs. 83.8% in non-AG; *p* = 0.204).

**Conclusion:**

First-line ART-failure exhibits high-level NRTI-resistance, with potential lower-efficacy of AZT compared to TDF. Significantly, using our 80% efficacy-threshold, only patients without NRTI-substitution on first-line could effectively switch to SLC following the WHO-approach. Patients with multiple NRTI-substitutions (exposed to both thymidine-analogues and TDF) on first-line ART would require HIVDR-testing to select active NRTIs for SLC.

**Electronic supplementary material:**

The online version of this article (10.1186/s12879-019-3871-0) contains supplementary material, which is available to authorized users.

## Background

By the end of 2017, about 70% of HIV-infected people knew their status, 77% of these were receiving antiretroviral therapy (ART) and 82% of treated patients achieved viral suppression [1, 2]. This progress led to 54% coverage of ART (14 million people) in Africa, with a coverage that is expected to double by 2020 as per the 90–90 − 90 targets (90% of all people living with HIV have been diagnosed; 90% of all people with diagnosed HIV infection are receiving ART; 90% of all people receiving ART have suppressed viral load) [2].

A major setback in achieving viral suppression is the emergence of HIV drug resistance (HIVDR), which is driven by delayed detection of virological failure and accumulation of drug resistant mutations (DRMs) in resource-limited settings (RLS). This later increases cases of new HIV-infections with resistant viruses, as well as AIDS-associated-morbidity and mortality [2, 3]. While HIVDR is gradually under control in high-income countries, the resistant patterns are rising in RLS and especially within sub-Saharan Africa (SSA) [3]. In these settings, one in every 10 adults starting ART harbour resistant virus; three in every 10 adults restarting first-line ART (i.e. women exposed to antiretrovirals for the prevention of mother-to-child transmission (PMTCT) or individuals re-initiating first-line ART after a period beyond 3 months of treatment interruption) harbour resistant virus; and five in every 10 young children diagnosed with HIV harbour resistant virus [3]. Regarding adults living in different SSA regions, resistance rates to first-generation non-nucleoside reverse-transcriptase inhibitors (NNRTIs), i.e. nevirapine and efavirenz, are already beyond 10% in East and Southern Africa regions [4], thus requiring treatment initiation with NNRTI-sparing regimens for optimal ART response in these settings, compared to West and Central Africa regions where the burden may still be bearable [4, 5]. However, the estimated incremental annual increase of 17% pre-treatment drug resistance (PDR) in West and Central Africa settings over time (*p* = 0.0017), the implementation of the “treat-all” strategy and the continuous use of NNRTI-based first-line ART [2], virological failure (VF) and acquired HIVDR to current first-line ART would be cumulative [5, 6], especially for countries like Cameroon where the national estimates of NNRTI-PDR are near the 10% critical threshold: 7.8% by Fokam et al. in 2017 and 8.3% by WHO in 2017 [7, 8].

With the complete phased-out of stavudine (D4T) from current first-line ART in RLS, tenofovir (TDF)-containing regimens are now widely used while patients with contraindication to TDF are generally being prescribed zidovudine (AZT)-containing regimens as an alternative [9, 10].

After VF on a standard first-line ART in RLS, an optimal selection of SLC is challenging due to limited access to HIVDR testing [2, 5, 6]. In such situations, 3TC could be recycled as part of the NRTI-backbone in SLC because it selects for the M184 V mutation, which decreases viral replication and increases susceptibility to AZT and TDF for potential use in SLC with PI/r [10, 11]. In this frame, the WHO guidelines also recommend the use of AZT + 3TC as NRTI backbone for SLC for those patients failing on TDF-containing first-line regimen; likewise, TDF + 3TC is recommended as NRTI backbone in SLC for patients failing on AZT-containing first-line regimen [9, 11]. Of note, failure on TDF leads to viral hyper-susceptibility to AZT (due to K65R mutation) while failure on AZT leads to possible cross-resistance to TDF. Moreover, in routine clinical practice, patients may experience several NRTI substitutions on first-line, which renders difficult drug recycling for use alongside PI/r in SLC. In an attempt to predict acquired HIVDR, Rutstein et al. reported a very poor sensitivity (14.7–28.0%) by using a risk-score approach [12]. Another report from South-India revealed that only half of the patients could be eligible for recycling NRTIs in the SLC [13], which somehow indicate an increasing need of implementing HIVDR testing in routine clinical practice of RLS [14]. However, Phillips et al. reported that HIVDR testing, as part of the decision to switch to SLC, was not cost-effective [15], thereby calling for the need of affordable HIVDR testing assays to the better management of ART in RLS [13–15]. A study in 9 countries revealed varying resistance patterns at first-line failure and consistent dual-class resistance [16], which raise concerns on using HIVDR testing in deciding to switch to SLC in RLS in the frame of increasing assess to reference laboratory platforms [15, 16]. Therefore, for a switch to SLC, assessing the potential effectiveness of the current WHO recommendation in real-life would be of great benefit for ART management in RLS.

Considering the peculiarity of the national ART program in Cameroon (scale-up of ART since 2003, generalised HIV epidemic, broad viral genetic diversity) [17, 18], we sought to evaluate the patterns of DRMs according to treatment history of patients failing first-line ART in routine clinical practice and the potential susceptibility of NRTIs for selecting active SLC.

## Methods

### Study design and settings

A cross-sectional and analytical study was conducted in 2016 among 101 HIV-infected patients failing first-line ART (defined as viral load: ≥ 1000 HIV-1 RNA copies/ml) and referred for HIVDR testing at the Chantal BIYA International Reference Centre (CIRCB) for research on HIV/AIDS prevention and management (CIRCB), located in Yaoundé, the capital city of Cameroon.

The CIRCB is a government institution of the Ministry of Public Health dedicated to HIV research and patient monitoring in several aspects, among which: (a) HIV early infant diagnosis in the frame of the national PMTCT program; (b) diagnosis of co-infections with HIV; (c) viral load measurement; (d) CD4 and CD8 T lymphocytes counts; (e) biochemical and haematological tests for drug safety; (f) HIV-1 genotypic drug resistance testing at subsidised costs; with a quality control program conducted in partnership with QASI (http://www.circb.cm/btc_circb/web/).

### RNA extraction, reverse transcription and polymerase chain reaction

Viral RNA was extracted from plasma samples using the QIAmp viral RNA Mini kit (Qiagen Hilden, Germany) according to the manufacturer’s protocol; extracted viral RNA was then reverse transcribed, amplified and sequenced as previously described [19]. Briefly, RNA was retro-transcribed and amplified using the kit One-Step Invitrogen SuperScript for long templates RT-PCR (Foster City, CA) and 2 sequence-specific primers for 40 cycles; for insufficiently amplified samples after the first round PCR, a second round PCR (semi-nested PCR) was performed; direct sequencing reaction was then carried out using 7 overlapping primers [19]; Capillary electrophoresis was performed using an Applied Biosystems 3130 XL genetic analyzer (Applied Biosystems, Tokyo, Japan) and sequences were assembled using SeqScape Version 2.7 to generate contigs [20].

Nucleotide sequences were aligned with subtype/circulating recombinant form (CRF) reference sequences from the Los Alamos National Laboratory database using the CLUSTALW integrated into Bioedit.7.2.5 [21, 22]. Following comparison of each sequence with reference sequences (database accessed on 8/01/2018) [23], gaps were then removed from the final alignment, and the phylogenetic tree was constructed by using Splitstree [24]. Recombination among HIV-1 subtypes were confirmed by SCUEAL [25], COMET [26], SimPlot [27], and Rega subtyping tool v.3 [28].

### Interpretation of drug resistance mutations

DRMs of the protease-reverse transcriptase regions were analyzed using the Stanford HIVDR database algorithm version 8.1 [29]. PI/r, NRTIs and NNRTIs effectiveness were interpreted using the genotypic scoring system for drug susceptibility with the following penalty: ≥60 high-resistance; 20–59: intermediate-resistance; < 20: susceptible. Resistance profile was then compared according to first-line ART-regimens received by each patient, and the potential drug efficacy was evaluated for SLC.

### Statistical analysis

Levels of DRMs, defined as any mutation with a genotypic penalty score of either a high or intermediate threshold, were used to classify patients failing ART with HIVDR. Per group (A, B and C), adequacy for using AZT versus TDF in SLC was defined as ≥80% of patients reporting drug efficacy based on a genotypic susceptibility profile within each group. Comparison of DRMs was performed by group (A, B and C) and following the local HIV-1 molecular epidemiology (CRF02_AG versus non-CRF02_AG). Chi-squared or Fisher’s exact test, where appropriate, was used for statistical analysis of categorical variables to determine the statistical significance of bivariate analysis, with *p* < 0.05 considered statistically significant.

### Ethical considerations

Ethical clearance was obtained from the Ethics Review and Consultancy Committee of the Cameroon Bioethics Initiative (Reference number: CBI/2013/0139/ERCC/CAMBIN), and written informed consent was provided. For those participants under 16 years of age, a written proxy-informed consent was provided to the study coordinators by the respective parent(s) or legal guardian(s), followed by an assent provided by the corresponding participant. Data were de-identified for purpose of confidentiality and privacy in data management.

## Results

### Characteristics of the study population

All study participants were failing treatment on a first-line ART available in the national guidelines, after a median time-on-ART of [IQR] 4 [2–5] years.

All the participants were patients exposed to lamivudine plus efavirenz or nevirapine, plus at least one other NRTIs used in the first-line ART of Cameroon. According to NRTI-exposure on first-line ART, patients were then classified into three groups (group-A: patients with prior but not concomitant exposure to both thymidine analogues AZT “and” D4T; group-B: exposed to TDF + a thymidine analogue D4T “or” AZT, and group-C: exposed solely to D4T), as shown in Table 1.

**Table 1:** Demographic and clinical data of patients

Total number of patients	101
Sex distribution	55.4% (56/101) male
Median age [IQR]	34 [10–41] years
Median CD4 [IQR]	129 [29–466] cells/μl
Median viral load [IQR]	71,630 [19,041-368,000] copies/ml
ART regimens received by each group of patients, in addition to 3TC plus EFV or NVP	
Group-A (*n* = 55)	both AZT and D4T
Group-B (*n* = 22)	TDF + “D4T or AZT”
Group-C (*n* = 24)	D4T (i.e. Triomune)

Legend. *3TC* Lamivudine, *EFV* Efavirenz, *NVP* Nevrapine, *ART* antiretroviral therapy, *AZT* Zidovudine, *D4T* Stavudine, *TDF* Tenofovir, *Triomune* D4T + 3TC + NVP. All patients had received 3TC plus EFV or NVP. Footnote: Prior exposure to D4T and AZT was not concomitant

### HIV drug resistance according to first line ART exposure

Globally, the rate of HIVDR among these patients failing first-line ART was 89.1% (90/101). Interestingly, up to 83.2% of patients harboured the M184 V mutation, associated with high-level resistance to 3TC and FTC and serving as adherence marker.

In all the three groups of ART-exposure, the overall prevalence of DRMs (both high and intermediate levels combined) to AZT was higher compared to TDF, with respectively: 56.4% (31/55) vs. 29.1% (16/55) in group A, *p* = 0.0038; 36.4% (8/22) vs. 31.8% (7/22) in group B, *p* = 1.000; and 62.5% (15/24) vs. 16.7% (4/24) in group C, *p* = 0.0032. This represents proportion of patients that have compromised AZT and/or TDF prior to switching to SLC, according to their ART history and without access to HIVDR testing.

Following class specific mutations, the prevalence of thymidine analogue mutations (TAMs) was higher in group-A and group-C combined (62.0% [49/79]) compared to group-B (40.9% [9/22]), *p* = 0.0765, leading to higher rates of HIVDR to AZT compared to TDF (Table 2 Group A, B and C). TDF-mutation K65R was significantly associated with TDF-exposure: 0% in group-A, 22.7% (5/22) in group-B, 4.2% (1/24) in group-C (*p* = 0.0013). Only 1.98% (2/101) of patients harboured PI/r major DRMs, in accordance with the history of non-exposure to PI/r.

**Table 2:** Resistance to each NRTI among patients failing first-line antiretroviral therapy

	Group-A: AZT and D4T *n* = 55		
HIVDR	High (%)	Intermediate (%)	Susceptible (%)
3TC	45 (81.8%)	0 (0.0%)	10 (18.2%)
ABC	11 (20.0%)	19 (34.5%)	25 (45.5%)
AZT	23 (41.8%)	8 (14.6%)	24 (43.6%)
D4T	23 (41.8%)	7 (12.7%)	25 (45.5%)
DDI	14 (25.4%)	11 (20.0%)	30 (54.6%)
FTC	45 (81.8%)	0 (0.0%)	10 (18.2%)
TDF	3 (5.5%)	13 (23.6%)	39 (70.9%)

	Group-B: TDF + D4T or AZT *n* = 22		
HIVDR	High (%)	Intermediate (%)	Susceptible (%)
3TC	19 (86.4%)	0 (0.0%)	3 (13.6%)
ABC	10 (45.5%)	6 (27.3%)	6 (27.3%)
AZT	3 (13.6%)	5 (22.7%)	14 (63.6%)
D4T	4 (18.2%)	9 (40.9%)	9 (40.9%)
DDI	10 (45.5%)	2 (9.1%)	5 (45.5%)
FTC	19 (86.4%)	0 (0.0%)	3 (13.6%)
TDF	6 (27.3%)	1 (4.5%)	15 (68.2%)

	Group-C: D4T (i.e. Triomune) *n* = 24		
HIVDR	High (%)	Intermediate (%)	Susceptible (%)
3TC	20 (83.3%)	0 (0.0%)	4 (16.7%)
ABC	7 (29.2%)	8 (33.3%)	9 (37.5%)
AZT	10 (41.7%)	5 (20.8%)	9 (37.5%)
D4T	10 (41.6%)	5 (20.8%)	9 (37.5%)
DDI	8 (33.3%)	4 (16.7%)	12 (50.0%)
FTC	20 (83.3%)	0 (0.0%)	4 (16.7%)
TDF	2 (8.3%)	2 (8.3%)	20 (83.3%)

Legend. *HIVDR* HIV drug resistance, *3TC* Lamivudine, *ABC* Abacavir, *AZT* Zidovudine, *D4T* Stavudine, *DDI* Didanosine, *FTC* Emtricitabine, *TDF* Tenofovir, *Triomune* D4T + 3TC + Nevirapine. Footnote: Prior exposure to D4T and AZT was not concomitant

### AZT and TDF potential efficacy according to treatment history after failing first-line ART

In group-A (i.e. exposed prior and not concomitantly to regimens containing both thymidine analogues AZT “and” D4T), the potential efficacy of AZT was significantly lower (43.64%) compared to that of TDF (70.91%); *p* = 0.0038.

In group-B (i.e. exposed prior and not concomitantly to TDF and a thymidine analogue AZT “or” D4T), the potential efficacy of AZT (63.64%) was similar to that of TDF (68.28%); *p* = 1.0000.

In group-C (i.e. exposure to D4T-containing regimen only), the potential efficacy of AZT was significantly lower (37.50%) compared to that of TDF (83.33%); *p* = 0.0032.

As shown in Table 2 Group A, B and C, the very high prevalence of resistance to 3TC and FTC were due to the high rate of M184 V mutation. Though not exposed to TDF, Groups A + C reported low prevalence of TDF-resistance, due to cross-resistance induced by the accumulation of other NRTI-mutations, especially TAMs. Of note, TAMs-1 were predominant (T215F/Y: 46.5%; M41 L: 22.8%; L210 W: 8.9%) and associated with higher levels of resistance to both AZT and TDF; as compared to TAMs-2 that had relatively lower prevalence (D67N: 21.8%; K70R: 19.8%; K219Q/E: 18.8%) and were associated preferentially with AZT/D4T-resistance.

### Genetic diversity of HIV-1 protease-reverse transcriptase

**Fig. 1:**
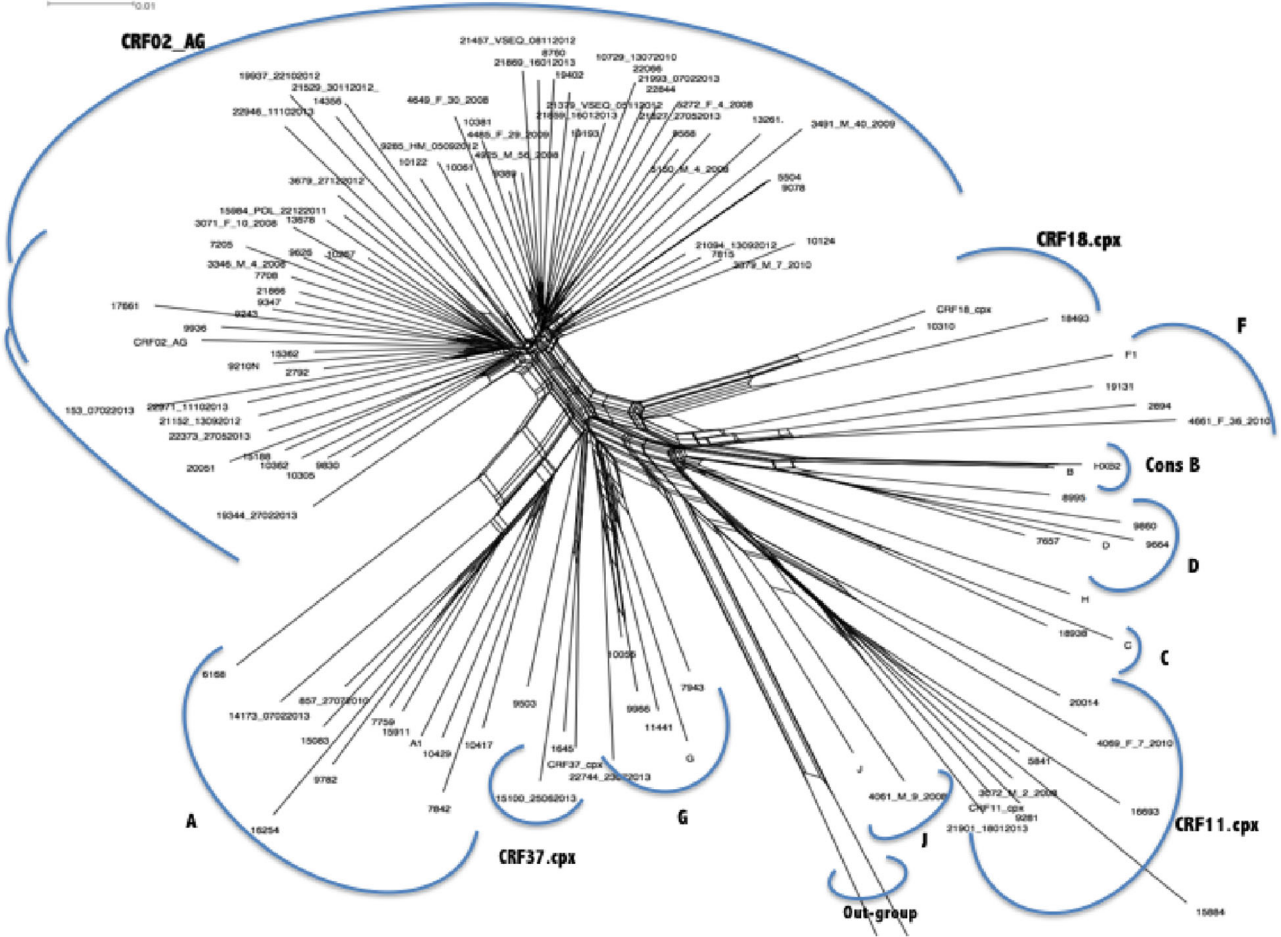
HIV-1 genetic diversity among patients failing first-line antiretroviral therapy in Cameroon. Legend. The reference sequences were from the Los Alamos Database (https://www.hiv.lanl.gov/components/sequence/HIV/search/search.html); Some references have been omitted to enable better visualization. The scale bar represents 1% genetic distance. CRF: circulating recombinant form

The genetic analysis revealed ten subtypes: 63.4% CRF02_AG (64/101), 8.9% CRF11.cpx (9/101), 7.9% A_1_ (8/101), 5.9% G (6/101), with only one case of subtype C known as an uncommon strain in west-central Africa (Fig. 1).

**Fig. 2:**
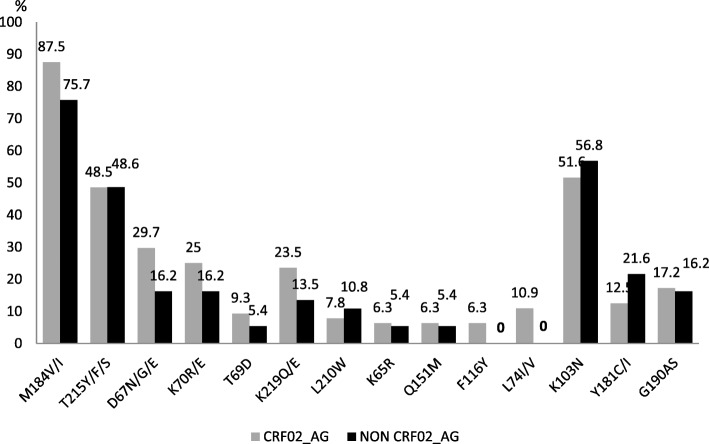
HIV-1 drug resistance mutations according to major subtypes distribution. Legend. CRF02_AG: circulating recombinant form; non-CRF02_AG: other subtypes other than CRF02_AG

According to the local HIV-1 molecular epidemiology, rate of DRMs was slightly higher in CRF02-AG (92.2%) compared to non-02_AG clades (83.8%), without any statistically significant difference (*p* = 0.204). Of note, L74I (following exposure to TDF-containing regimens) and L210 W (following exposure to regimens containing a thymidine analogue) mutations were found only in the group of patients infected with CRF02_AG viruses (Fig. 2). HIVDR by drug-class also revealed similar rates between CRF02_AG and non-CRF02_AG infected populations (Tables 3 and 4).

**Table 3:** Prevalence of HIV-1 drug resistance among CRF02_AG

Resistance Category	No. sequences	Percentage with DRM	1 DRM	2 DRMs	3 DRMs	≥4 DRMs
PI/r	64	1.6%	1	0	0	0
NRTI	64	87.5%	10	12	12	22
NNRTI	64	92.2%	33	20	6	0

Legend. *PI/r* ritonavir boosted protease inhibitor, *NRTI* nucleos(t) ide reverse transcriptase inhibitor; *NNRTI* non-nucleoside reverse transcriptase inhibitor, *DRM* drug resistance mutations

**Table 4:** Prevalence of HIV-1 drug resistance among non-CRF02_AG

Resistance Category	No. sequences	Percentage with DRM	1 DRM	2 DRMs	3 DRMs	≥4 DRMs
PI/r	37	2.7%	0	0	0	1
NRTI	37	75.7%	7	6	5	10
NNRTI	37	83.8%	19	10	2	0

## Discussion

With the limited access to HIVDR testing in RLS, successful switch to SLC remains a major clinical challenge, especially for patients heavily treated on first-line ART (i.e. substitution of several NRTIs) [2, 5, 6]. Thus, implementing local strategies to ensure a successful switch to SLC is warranted [10].

With a median duration of 4 years on ART, the severe immunodeficiency (CD4 < 200 cells/mm^3^) and the high viral load (HIV-RNA > 10.000 copies/ml), there is a late detection of treatment failure and a substantial accumulation of DRMs in about nine out of ten patients in routine care [12–14, 17]. This observation therefore urges the need for early viral load monitoring for timely detection of ART failure and adequate switch to SLC with limited risk of HIVDR emergence [30–32]. Our findings are similar to several reports in Cameroon [31, 32], but with higher HIVDR prevalence compared to a study conducted at 36-months ART [33]. This is due to differences in study design (virologically suppressed and unsuppressed patients) and durations [33].

Most importantly, with only ~ 2% PI/r resistance, the use of PI/r as back bone for SLC remains standard for patients failing first-line regimens in settings with similar ART programs [2, 4, 9, 11], pending the selection of potentially active NRTIs [10–14, 16].

In group-A (both AZT + D4T-exposure), level of HIVDR to AZT was almost two times higher as compared to TDF. This could be explained by the fact that these patients were previously exposed to D4T-containing regimens (i.e. *Triomune*) and were subsequently moved to AZT, most likely due to D4T-adverse events or the phased-out of D4T [34]. In the frame of treatment failure, the accumulation of TAMs would further jeopardise the efficacy of TDF due to cross-resistance mainly driven by TAMs-1 [34, 35]. Therefore, among patients exposed to both thymidine analogues, TDF still stands as the preferable option despite risks of TAMs-induced cross-resistance (~ 30%). Thus, in routine clinical practice, patients failing ART with such treatment history should either: (a) be referred for HIVDR testing or (b) be switched blindly to SLC with TDF under close viral load monitoring to detect those at risk of failure due to TDF cross-resistance [30, 34].

In group-B (exposure to TDF + a thymidine analogue D4T “or” AZT), about one-third of patients harboured HIVDR to AZT, also similar for TDF (*p* = 1.0000). This is due to the selection of DRMs (TAMs and/or K65R) following previous ART with a thymidine analogue- and subsequently with TDF-containing regimens [35]. Thus, because one-third of patients managed on first-line with such NRTIs substitution has lost both TDF- and AZT-efficacy [30–34], HIVDR testing would be of great clinical relevance for selecting SLC NRTIs [14, 36].

In group-C (exposed only to a single regimen D4T-3TC-NVP), level of HIVDR was significantly higher to AZT as compared to TDF (about 4-folds), with minimal effects of TAMs inducing cross-resistance to TDF [20]. Thus, in case of exposure to a single first-line regimen, the use of TDF in SLC would have a high predictive efficacy in the majority of patients (≥80%). Thus, for such patients living in RLS, using TDF in SLC without referring to HIVDR testing might be acceptable in clinical practice [15].

As expected, K65R was only found from the group of TDF-exposed patients (group-B). Of note, K65R is a mutation known to reverse the excision phenotype of AZT resistance mutations, to increase viral susceptibility to AZT, which in turns improves the clinical efficacy of AZT [10, 37]. Thus, a wider use of TDF in current first-line regimen might improve the efficacy of AZT for subsequent use in SLC. However, the poor rate of AZT-efficacy in this group is attributed to TAMs derived from previous exposure to thymidine analogues (AZT or D4T). This implies that without HIVDR testing, AZT could be used in SLC solely for those failing on TDF-containing regimens without any previous substitution of NRTIs [8–11].

The very high frequency of M184 V (> 80%) underscores the utility of this mutation as an indicator of therapeutic compliance in clinical practice for patients receiving any regimen containing 3TC or FTC [9]. Though FTC has an enhanced incorporation efficiency (~ 10-fold) compared to 3TC during HIV-1 RT-catalyzed RNA-dependent DNA synthesis [38], these two cytidine analogues appear as suitable alternatives without the need for programme-wide substitution of FTC for 3TC in current clinical practice [39, 40].

Phylogeny confirms CRF02_AG as the major circulating clade [41–45]. The single case of subtype C (uncommon in Cameroon) may be due to phylodynamics from Southern/Eastern Africa [46, 47]. Even though the analysis of CRF02_AG versus non-AG showed no major effect of the local subtype distribution on emerging DRMs, molecular epidemiology surveillance merits further investigations, which include the preferential pattern of L74I as a potential signature in CRF02_AG-infected patients [48, 49]. Findings with clinical responses after switch to SLC would provide greater insights for translational application [50].

## Conclusion

In a nutshell, first-line ART-failure exhibits variable levels of NRTI-resistance (from 17 to 62.5%) in a routine clinical setting of Cameroon, with a remarkable higher level of AZT-resistance as compared to TDF-resistance (recently introduced drug). Thus, regarding efficacy, first-line ART-failure without NRTI-substitution (i.e. exposed to only AZT or TDF) on first-line could switch to SLC following the WHO-approach. However, failure after substitution only between thymidine analogues may receive TDF as part of SLC pending a close viral load or HIVDR-testing whenever possible. Most importantly, failure after multiple NRTI-substitutions on first-line (exposed to both “thymidine-analogue and TDF”) should be referred for HIVDR-testing for selecting active NRTIs for an optimal SLC in clinical practice.

## Additional files

Additional file 1: SMF1 - accession numbers. (TXT 33 kb)

Additional file 2: SMF2 - GenBank Submissions grp 6,957,604. (TXT 90 kb)

## Abbreviations

ART: Antiretroviral therapy,
CIRCB: Chantal BIYA International Reference Centre for research on HIV/AIDS prevention and management, DNA Deoxyribonucleic acid,
DRM: Drug resistance mutations,
HIV: Human immunodeficiency virus,
HIVDR: HIV drug resistance,
IQR: Interquartile range,
NNRTI: Non-Nucleoside reverse transcriptase inhibitor,
NRTI: Nucleos(t) ide reverse transcriptase inhibitor,
PLHIV: People living with HIV,
RLS: Resource-limited settings,
RNA: Ribonucleic acid,
SLC: Second-line combination,
SSA: Sub-Saharan Africa,
TAM: Thymidine analogue mutation,
UNAIDS: United Nations program on AIDS,
WHO: World health organisation

## References

[1] World Health Organization. HIV/AIDS: data and statistics*.* Geneva: World Health Organization; 2018 http://www.who.int/hiv/data/en/, (Accessed on 09 January 2018).

[2] World Health Organization. Global Health sector strategy on HIV, 2016–2021*.* Geneva: World Health Organization; 2017*.* http://www.who.int/hiv/strategy2016-2021/en/, (Accessed on 09 January 2018).

[3] World Health Organization (2018). Progress report. Global action plan on HIV drug resistance 2017–2021.

[4] Hamers RL, Wallis CL, Kityo C (2011). HIV-1 drug resistance in antiretroviral-naive individuals in sub-Saharan Africa after rollout of antiretroviral therapy: a multicentre observational study. Lancet Infect Dis.

[5] Günthard HF, Calvez V, Paredes R, et al. Human immunodeficiency virus drug resistance: 2018 recommendations of theInternational antiviral society-USA panel. Clin Infect Dis. 2018. 10.1093/cid/ciy463. 10.1093/cid/ciy463 PMC6321850 30052811

[6] World Health Organization. Policy brief. Transition to new antiretrovirals in HIV treatment programs*.* Geneva: World Health Organization; 2017. http://www.who.int (Accessed on 09 January 2018).

[7] Fokam J, Santoro MM, Takou D, et al. Pre-treatment HIV-1 drug resistance and genetic diversity in different regions of Cameroon: the CIRCB country-survey. In: Proceedings of the XXVI international workshop on HIV drug resistance and treatment strategies. Johannesburg. South-Africa, 6-8 November 2017.

[8] World Health Organization. Guidelines on the public health response to pretreatment HIV drug resistance*.* Geneva: World Health Organization; 2017 (http://www.who.int/hiv/pub/guidelines/hivdr-guidelines-2017/en, accessed 26 June 2018).

[9] World Health Organisation. Consolidated guidelines on the use of antiretroviral drugs for treating and preventing HIV infection: Recommendations for a public health approach. Second edition. Geneva: 2016. http://www.who.int (Accessed on 09 January 2018). 27466667

[10] Dinesha TR, Gomathi S, Boobalan J (2016). Genotypic HIV-1 drug resistance among patients failing Tenofovir-based first-line HAARTin South India. AIDS Res Hum Retrovir.

[11] Pillay D, Albert J, Bertagnolio S (2013). Implications of HIV drug resistance on first- and second-line therapies in resource-limited settings: report from a workshop organized by the collaborative HIV and anti-HIV drug resistance network. Antivir Ther.

[12] Rutstein SE, Hosseinipour MC, Weinberger M (2016). Predicting resistance as indicator for need to switch from first-line antiretroviral therapy among patients with elevated viral loads: development of a risk score algorithm. BMC Infect Dis.

[13] Sivamalar S, Dinesha TR, Gomathi S (2017). Accumulation of HIV-1 drug resistance mutations after first-line immunological failure to evaluate the options of recycling NRTI drugs in second-line treatment: a study from SouthI ndia. AIDS Res Hum Retrovir.

[14] Lessells RJ, Avalos A, de Oliveira T (2013). Implementing HIV-1 genotypic resistance testing in antiretroviral therapy programs in Africa: needs, opportunities, and challenges. AIDS Rev.

[15] Phillips A, Cambiano V, Nakagawa F (2014). Cost-effectiveness of HIV drug resistance testing to inform switching to second line antiretroviral therapy in low income settings. PLoS One.

[16] Harrison L, La Rosa A, Viana RV et al. Is resistance testing of value after first-line art failure in resource-limited settings? - insights from ACTG 5273. Proceedings of the XXV international workshop on HIV drug resistance and treatment strategies, Boston, Massachusetts, USA, February 20–21, 2016.

[17] Billong SC, Fokam J, Aghokeng AF (2013). Population-based monitoring of emerging HIV-1 drug resistance on antiretroviral therapy and associated factors in a sentinel site in Cameroon: low levels of resistance but poor programmatic performance. PLoS One.

[18] Fokam J, Billong SC, Bissek AC (2013). Declining trends in early warning indicators for HIV drug resistance in Cameroon from 2008-2010: lessons and challenges for low-resource settings. BMC Public Health.

[19] Fokam Salpini R, Santoro MM (2011). Performance evaluation of an in-house human immunodeficiency virus type-1 protease-reverse transcriptase genotyping assay in Cameroon. Arch Virol.

[20] Technologies, L., SeqScape Software 3*.*https : // tools.thermofisher.com / content / sfs / manuals / 4474242A.pdf, 2012.

[21] Thompson JD, Higgins DG, Gibson TJ (1994). CLUSTAL W: improving the sensitivity of progressive multiple sequence alignment through sequence weighting, position-specific gap penalties and weight matrix choice. Nucleic Acids Res.

[22] Hall T (2011). Bioedit: an important software for molecular biology. GERF Bull Biosci.

[23] LANL., *Consensus Maker. .*http : // www.hiv.lanl.gov / content / sequence / CONSENSUS / consensus.html 2015.

[24] Huson DH, Bryant D (2006). Application of phylogenetic networks in evolutionary studies. Mol Biol Evol.

[25] Kosakovsky Pond SL (2009). An evolutionary model-based algorithm for accurate phylogenetic breakpoint mapping and subtype prediction in HIV-1. PLoS Comput Biol.

[26] Struck D (2014). COMET: adaptive context-based modeling for ultrafast HIV-1 subtype identification. Nucleic Acids Res.

[27] Lole KS (1999). Full-length human immunodeficiency virus type 1 genomes from subtype C-infected seroconverters in India, with evidence of intersubtype recombination. J Virol.

[28] Pineda-Pena AC (2013). Automated subtyping of HIV-1 genetic sequences for clinical and surveillance purposes: performance evaluation of the new REGA version 3 and seven other tools. Infect Genet Evol.

[29] Liu TF, Shafer RW (2006). Web resources for HIV type 1 genotypic-resistance test interpretation. Clin Infect Dis.

[30] Kamalendra Singh JAF, Kirby KA, Neogi U, Sonnerborg A, Hachiya A, Das K, Arnold E, McArthur C, Parniak M, Sarafianos SG (2014). Drug resistance in non-B subtype HIV-1: impact of HIV-1 reverse transcriptase inhibitors. Viruses.

[31] Fokam J, Salpini R, Santoro MM (2011). Drug resistance among drug-naive and first-line antiretroviral treatment-failing children in Cameroon. Pediatr Infect Dis J.

[32] Ceccarelli L, Salpini R, Moudourou S (2012). Characterization of drug resistance mutations in naïve and ART-treated patients infected with HIV-1 in Yaounde, Cameroon. J Med Virol.

[33] Aghokeng AF, Kouanfack C, Eymard-Duvernay S (2013). Virological outcome and patterns of HIV-1 drug resistance in patients with 36 months’ antiretroviral therapy experience in Cameroon. J Int AIDS Soc.

[34] Kuritzkes DR, Bassett RL, et al. Rate of thymidine analogue resistance mutation accumulation with zidovudine- or Stavudine-based regimens. J Acquir Immune Defic Syndr. 2004;36. 10.1097/00126334-200405010-00008. 10.1097/00126334-200405010-00008 15097303

[35] World Health Organisation (2013). Consolidated guidelines on the use of antiretroviral drugs for treating and preventing HIV infection: recommendations for a public health approach.

[36] Luber AD (2005). Genetic barriers to resistance and impact on clinical response. MedGenMed.

[37] White KL, Chen JM, Feng JY (2006). The K65R reverse transcriptase mutation in HIV-1 reverses the excision phenotype of zidovudine resistance mutations. Antivir Ther.

[38] Feng JY, Shi J, Schinazi RF, Anderson KS (1999). Mechanistic studies show that (−)-FTC-TP is a better inhibitor of HIV-1 reverse transcriptase than 3TC-TP. FASEB J.

[39] McColl DJ, Margot N, Chen SS (2011). Reduced emergence of the M184V/I resistance mutation when antiretroviral- naive subjects use emtricitabine versus lamivudine in regimens composed of two NRTIs plus the NNRTI efavirenz. HIV Clin Trials.

[40] Tang MW, Kanki PJ, Shafer RW (2012). A review of the virological efficacy of the 4 World Health Organization-recommended tenofovir-containing regimens for initial HIV therapy. Clin Infect Dis.

[41] Nanfack AJ, Redd AD, Bimela JS (2017). Multimethod longitudinal HIV drug resistance analysis in antiretroviral-therapy-naive patients. J Clin Microbiol.

[42] Agyingi L, Mayr LM, Kinge T (2014). The evolution of HIV-1 group M genetic variability in southern Cameroon is characterized by several emerging recombinant forms of CRF02_AG and viruses with drug resistance mutations. J Med Virol.

[43] Fokam J, Takou D, Santoro MM (2016). Short communication: population-based surveillance of HIV-1 drug resistance in Cameroonian adults initiating antiretroviral therapy according to the World Health Organization guidelines. AIDS Res Hum Retrovir.

[44] Courtney CR, Agyingi L, Fokou A (2016). Monitoring HIV-1 group M subtypes in Yaounde, Cameroon reveals broad genetic diversity and a novel CRF02_AG/F2 infection. AIDS Res Hum Retrovir.

[45] Teto G, Tagny CT, Mbanya D (2017). Gag P2/NC and pol genetic diversity, polymorphism, and drug resistance mutations in HIV-1 CRF02_AG- and non-CRF02_AG-infected patients in Yaoundé, Cameroon. Sci Rep.

[46] Mir D, Jung M, Delatorre E (2016). Phylodynamics of the major HIV-1 CRF02_AG African lineages and its global dissemination. Infect Genet Evol.

[47] Manasa J, Danaviah S, Lessells R (2016). Increasing HIV-1 drug resistance between 2010 and 2012 in adults participating in population-based HIV surveillance in rural KwaZulu-Natal, South Africa. AIDS Res Hum Retrovir.

[48] Miller MDM, Damian MC, Cheng AK (2007). K65R development among subtype C HIV-1-infected patients in tenofovir DF clinical trials. AIDS Res Hum Retrovir.

[49] Kaleebu P, French N, Mahe C (2002). Effect of human immunodeficiency virus (HIV) type 1 envelope subtypes a and D on disease progression in a large cohort of HIV-1-positive persons in Uganda. J Infect Dis.

[50] Fokam J, Bellocchi MC, Armenia D (2018). Next-generation sequencing provides an added value in determining drug resistance and viral tropism in Cameroonian HIV-1 vertically infected children. Medicine.

